# Severe Neutropenia Complicated with Necrotizing Fasciitis Unveils a Diagnosis of Rheumatoid Arthritis: A Case Report

**DOI:** 10.7759/cureus.4079

**Published:** 2019-02-15

**Authors:** Dima Nimri, Mohamed A Abdallah, Qazi Ahmed Waqas, Abdelmohaymin Abdalla, Hamza Tantoush

**Affiliations:** 1 Internal Medicine, University of South Dakota, Sioux Falls, USA

**Keywords:** rheumatoid arthritis, felty syndrome, neutropenia

## Abstract

Felty syndrome, a rare extra-articular manifestation of rheumatoid arthritis (RA), usually affects patients with long-standing disease. Patients with this syndrome typically present with neutropenia, splenomegaly, in addition to erosive RA. The development of unexplained neutropenia in healthy patients should prompt the work up for Felty syndrome, especially in patients with suggestive demographics, signs, and symptoms. Differentiation between large granular lymphocyte (LGL) leukemia and Felty syndrome is necessary as both can present with neutropenia, and are associated with RA. Immunosuppressive therapy has improved the prognosis of patients with Felty syndrome given the decreasing rates of splenectomies done in those patients over the last decades.

## Introduction

Felty syndrome is one of the rare, severe extra-articular manifestations of rheumatoid arthritis (RA) [1]. The triad of neutropenia, splenomegaly, and RA is characteristic [1]. It is reported in less than one percent of patients with RA [2]. Typically, Felty syndrome tends to affect patients with long-standing erosive RA [2]. The diagnosis of Felty syndrome as the first manifestation of RA is exceedingly uncommon [2]. Here, we present a case of severe neutropenia in an otherwise healthy asymptomatic female patient. Her presentation uncovered a diagnosis of Felty syndrome.

## Case presentation

A 64-year-old Caucasian female with no significant past medical history presented to the emergency department with right upper extremity pain, redness, and blistering that started seven days prior to presentation. The patient denied history of trauma, fever, or night sweats, but reported progression of symptoms. She reported having mild generalized joint pain for at least five years. The patient denied also any history of joint swelling, redness or warmth, dry eyes, skin nodules, rashes or lesions, or personal or family history of RA or autoimmune disease.

On physical examination: vital signs were stable and body mass index was 25.7 kg/m2. There was evidence of diffuse redness, blistering and swelling over her right arm, elbow joint and forearm, with marked tenderness over the right arm. It was noted that the patient had mild bilateral ulnar deviation, but no evidence of joint tenderness, swelling, or redness of the small joints of the hands.

 Her initial lab work included a complete blood count with a white blood cell (WBC) count of 0.9 (Ref: 4-11 × 103 cells/ul) with an absolute neutrophil count (ANC) of 387 (Ref: 1.8-0.8 × 103 cells/ul), platelet count of 109,000 (Ref: 140-400 × 103 platelets/ul) with normal hemoglobin of 13.3 g/dl (Ref: 11.5-15.8 g/dl). Initial right upper extremity radiographs did not show evidence fractures, but did show soft tissue swelling over the right forearm and the right elbow joint. Initial suspicion was for superficial cellulitis and olecranon bursitis. However, further evaluation with right upper extremity duplex showed evidence of acute deep venous thrombosis (DVT) of the brachial vein. The patient was started on intravenous (IV) heparin and IV vancomycin and cefepime empirically for the treatment of acute DVT and soft tissue infection and was subsequently admitted for further workup.

Over the next two days of hospitalization, the patient failed to show significant improvement in her symptoms. Right upper extremity magnetic resonance imaging (MRI) with contrast showed superficial and deep fasciitis with myonecrosis over the right arm. Necrotizing fasciitis was suspected. The patient underwent emergent surgical exploration with irrigation and debridement that showed an evidence of deep fasciitis. A repeat surgical exploration was done two days later. Bacterial cultures grew methicillin-sensitive *Staphylococcus aureus* and group C streptococcus. IV Clindamycin and cefepime were continued. She was switched to oral clindamycin after obtaining antibiotics sensitivity results.

Regarding the patient’s neutropenia and thrombocytopenia, serology results for hepatitis A and B, cytomegalovirus (CMV) and human immunodeficiency virus (HIV) were obtained and came back negative. The patient underwent a bone marrow biopsy, which revealed a hypercellular bone marrow with increased megakaryocytes and CD8 positive cells. Flow cytometry was negative for lymphoproliferative disease. Her T-cell receptor rearrangement by polymerase chain reaction was positive. Her ANC continued to drop over the following days, despite the fact she received granulocyte colony-stimulating factor (G-CSF) for five days initially. Given the bilateral ulnar deviation and elevated C-reactive protein (CRP) on presentation, work up for Felty syndrome was initiated. Labs showed a CRP of 238.8 (0-5 mg/l) and erythrocyte sedimentation rate (ESR) of 78 (Ref: 0-22 mm/h). Her rheumatoid factor (RF) was positive at 24 (Ref: 0-11 IU/ml), anti-citrullinated protein antibodies (anti-CCP) of >340 (Ref: 0-6.9 U/ml), and antinuclear antibodies (ANA) of 1:320 (<1:40). She had negative anti-SSA and SSB antibodies as well as normal levels of C3 and C4. Hepatosplenomegaly was noted on computed tomography (CT) scan of the abdomen. Bilateral foot and hand X-rays revealed osteopenia, arthritis, and erosive changes in the metatarsophalangeal (MTP) and metacarpophalangeal (MCP) joints, respectively. A diagnosis of Felty syndrome was made. Oral prednisone 40 mg and methotrexate were initiated prior to hospital discharge. Her ANC improved dramatically from a low value of 40 to 1530 within two weeks of initiating treatment.

## Discussion

Rheumatoid arthritis is a chronic, inflammatory, polyarthritis that has many systemic manifestations. While the involvement of skin, eyes, lungs, and heart is typical, kidney, central and peripheral nervous system, and blood vessel involvement are less common [3]. Felty syndrome is a very rare extra-articular manifestation of seropositive RA. The majority of patients with Felty syndrome usually have severe, long-standing (more than 10 years), erosive arthritis with deformities [4]. Felty syndrome presenting as the initial presentation of RA, whether in patients with seronegative arthritis or in those without arthritis is extremely unusual [5-6]. Although our patient did not have an established diagnosis of RA, the presence of erosions in hand and feet radiographs may suggest the presence of subclinical RA.

The exact etiology is unknown. Patients with Felty syndrome are typically females in their fourth decade. Absolute neutropenia is present in all patients. It increases the risk of bacterial infections, namely, respiratory tract infections and skin and soft tissue infections [5]. Other manifestations of Felty syndrome are similar to extra-articular RA and include pleuropericarditis, vasculitis, rheumatoid nodules, lymphadenopathy, anemia, and thrombocytopenia [2-3, 5]. Patients with this syndrome typically have an elevated CRP and ESR, as well as positive anti-CCP antibodies. Other antibodies, such as anti-neutrophil cytoplasmic antibodies (ANCA), ANA, anti-DS, anti-histone antibodies, anti-glucose-6-phosphate isomerase antibodies may be present [7-8].

Diagnosis is made after exclusion of other causes of neutropenia. Of note, large granular lymphocyte (LGL) leukemia is an important differential diagnosis as it shares some features with Felty syndrome. Neutropenia, splenomegaly, an association with RA and HLA-DR4 are common in both syndromes. However, the presence of polyclonality on bone marrow biopsy in patients with Felty syndrome, as opposed to monoclonality seen in LGL leukemia can help differentiate between the two disease entities. This can be detected by testing for rearrangements of the T-cell receptor by polymerase chain reaction. In addition, immunophenotyping in LGL will indicate the presence of a population of cytotoxic T lymphocytes expressing a set of surface markers (e.g., CD 2, 3, 8, 16, and 57), which is infrequent in Felty syndrome [9-11]. However, differentiation between these two syndromes is often challenging. The term pseudo-Felty syndrome was used to label patients who were initially diagnosed with Felty syndrome, but afterwards found to have LGL leukemia. Our patient did not have any evidence of that.

Treatment of Felty syndrome should focus on restoration of neutropenia as that can decrease the risk of recurrent infection. Methotrexate therapy up to 25 mg weekly should be tried initially, with or without prednisone (up to 40 mg daily) as bridging therapy [4, 12]. Rituximab can be added for patients who did not respond to methotrexate therapy [13]. Tumor necrosis factor (TNF) inhibitors have failed to show clinical activity in patients with Felty syndrome [14]. In refractory patients, addition of Abatacept (a selective costimulation modulator that inhibits T-cells) can be tried before considering splenectomy [15]. Filgrastim or G-CSF can elevate the ANC quickly, and its use for the treatment of Felty syndrome can be justified in patients with severe neutropenia, who have life-threatening infections and/or in those who have poor response to initial therapy [16]. G-CSF can cause increased arthritis and vasculitis in some patients when the WBC counts increases [16-17]. Splenectomy is indicated in patients with severe, recurrent bacterial infections or chronic nonhealing leg ulcers refractory to treatment. However, neutropenia can recur in 25% of patients who undergo splenectomy [11, 17-18]. Prognosis of patients with Felty syndrome has improved significantly over the years since recognition of this syndrome. This is evident by decreased rates of hospitalization and rates of splenectomies as well [16].

## Conclusions

Patients with otherwise unexplained neutropenia should be considered for the diagnosis of Felty syndrome, a rare extra-articular manifestation of RA with severe consequences. Other important differential diagnoses which need to be considered include LGL leukemia, systemic lupus erythromatosis (SLE), and medications’ side effects. Association with RA, elevated acute phase reactants (CRP, ESR), and presence of raised anti-CCP antibodies are suggestive. A bone marrow examination is necessary for differentiation between Felty syndrome and LGL leukemia. Immunosuppressive therapy is usually focused on restoring neutrophil counts, preventing recurrent infections and underlying RA.
